# A rare inborn error of metabolism associated with a Dandy–Walker malformation

**DOI:** 10.1007/s13760-012-0098-x

**Published:** 2012-06-15

**Authors:** Limeme Manel, Zaghouani Ben Alaya Houneida, Amara Habib, Bakir Dejla, Kraiem Chekib

**Affiliations:** 1 Imaging Department of Farhat Hached Hospital, 4000 Sousse, Tunisia; 2 Rue de la liberté, immeuble Limame, App n°2, Sahloul 2, 4054 Sousse, Tunisia

## Observation

**Fig. 1:**
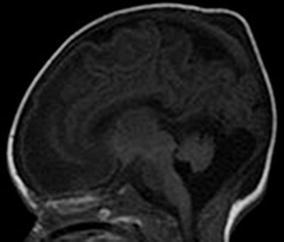
MR images. Sagittal spin-echo T1-weighted MR image reveals expansion of the cisterna magna communicating with the fourth ventricle, a cerebellar vermis and a corpus callosum hypoplasia

**Fig. 2:**
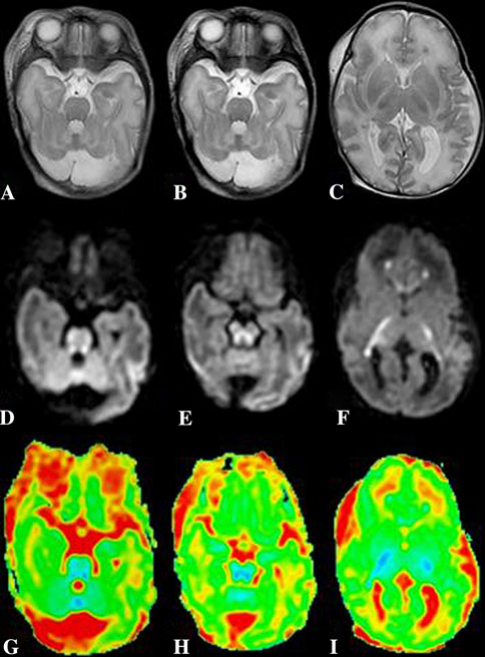
MR images. Axial fast spin-echo T2-weighted MR images show high signal intensity in the middle cerebellar peduncles (**a**), the dorsal brain stem (**b**), and the in the posterior limbs of the internal capsules (**c**) suggesting abnormal myelin. **d**, **e**, **f** Diffusion-weighted MR image demonstrates high signal intensity in these areas. **g**, **h**, **i** ADC map shows decreased ADC values in these areas consistent with restricted diffusion

**Fig. 3:**
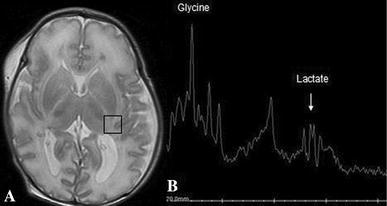
Proton MR imaging. **a** Axial T2-weighted image with the voxel location for proton MR spectroscopy. **b** Spectrum obtained from the parietal white matter shows a peak of glycine at 3.55 ppm and a lactate peak at 1.33 ppm

A baby girl, born to second-degree consanguineously married couple, was delivered by forceps, after a full-term pregnancy. Apgar scores were 9 and 10, birth weight 4,100 g, and head circumference 35 cm. The baby was hospitalized, at the age of 20 days, for recurrent myoclonic seizures. Physical examination found hypotonia and absent reflexes. Amino acid analysis of the cerebrospinal fluid (CSF) and plasma was performed, to exclude the possibility of metabolic disorder. A brain ultrasonography found hypoplastic corpus callosum. An expansion of the cisterna magna communicating with the fourth ventricle and a cerebellar vermis hypoplasia were also showed. These findings, suggested a Dandy–Walker malformation. 1 day later, brain magnetic resonance (MR) imaging and spectroscopy were performed. MRI confirmed the brain ultrasonography findings (Fig. 1). On the T1- and T2-weighted images, the appearance of the white matter myelination was inappropriate for age. The axial T2-weighted images revealed increased signal intensity in the dorsal brain stem, the middle cerebellar peduncles, and the posterior limbs of the internal capsules. Diffusion-weighted MR images found hyperintense signal intensity in these areas, in combination with low apparent diffusion coefficient (ADC) values, consistent with restricted diffusion (Fig. 2). Localized proton MR spectroscopy showed a markedly increased peak intensity at 3.55 ppm, which was assigned to glycine and a lactate peak at 1.33 ppm (Fig. 3). The diagnosis of nonketotic hyperglycinemia (NKH) was made by laboratory findings of elevated CSF and plasma glycine levels, which were 250 μmol/L (normal range 1–15 μmol/L) and 1,540 μmol/L (normal range 230–740 μmol/L), respectively. The CSF-to-plasma glycine ratio was also elevated to 0.16 (normal range 0.02–0.03).

4 days later, the baby became lethargic, and the shallow respiration which began at that point progressed to intermittent apnea, requiring mechanical ventilation. 1 day later the infant died.

## Discussion

Nonketotic hyperglycinemia is a rare autosomal recessive metabolic brain disorder caused by deficient activity of the glycine cleavage enzyme system, resulting in high glycine concentrations in urine, plasma, CSF, and brain [1, 2]. Such increases cause vacuolating myelinopathy in myelinated areas at birth [2–4].

Clinical manifestations are lethargy, hypotonia, epileptic seizures, apnea, and rapid progression to coma [1, 2]. Brain malformations in NKH include ventriculomegaly, dysgenetic corpus callosum, and posterior fossa cysts [3, 4]. Dandy–Walker malformation is a congenital disorder that involves the cerebellar vermis and fourth ventricle. It results in an induction failure of the opposing cerebellar plates, with persistence of the membranous area of the fourth ventricle [5].

T2-weighted MR images show increased signal intensity in myelinated areas: the ascending tracts in the brain stem, posterior limbs of the internal capsules, the cerebellar peduncles, optic tracts, and optic chiasm [2]. Diffusion-weighted MR images reveal restricted diffusion attributed to the accumulation of fluid between the layers of myelin lamellae [4]. MRS detects increased glycine concentration in the brain, peaking at 3.55 ppm. There is a close correlation between glycine levels and the clinical status of the patient. An increased lactate peak can be observed and may reflect anaerobic glycolysis of brain tissue, probably caused by hypoxia [1]. Diagnosis is made by showing high CSF and plasma glycine levels [3]. Most patients die during the neonatal period [2, 4].

The recognition of NKH leads not only to immediate care of the patient, but also to genetic counseling to prevent recurrences in further pregnancies [3].
